# 1960. Mucosal rAd5 Immunization against SARS-CoV-2 Spike Elicits Cross-Reactive Nasal and Serum Neutralizing Antibodies and Protects Against Beta Variant Challenge in African Green Monkeys

**DOI:** 10.1093/ofid/ofac492.1586

**Published:** 2022-12-15

**Authors:** Becca A Flitter, Sarah N Tedjakusuma, Colin A Lester, Elena D Neuhaus, Susan Johnson, Emery G Dora, Nadine Peinovich, Clara B Jegede, Sean N Tucker

**Affiliations:** Vaxart Inc, South San Francisco, California; Vaxart, South San Francisco, California; Vaxart, Inc., South San Francisco, California; Vaxart, South San Francisco, California; Vaxart, South San Francisco, California; Vaxart, South San Francisco, California; Vaxart, South San Francisco, California; Vaxart, South San Francisco, California; Vaxart, South San Francisco, California

## Abstract

**Background:**

Mucosal vaccination may offer increased protection against SARS-CoV-2 compared to parental immunization. Here, we describe immunogenicity and efficacy following viral challenge in non-human primates after intranasal delivery of three unique non-replicating adenoviral vector vaccine (rAd5) candidates.

**Methods:**

African green monkeys (AMG) were prime boost immunized 29 days apart with vaccine candidates either expressing the parental spike protein alone (Wuhan-S), spike plus nucleocapsid (Wuhan-S-N), or the spike protein from the beta variant (beta-S). Serum and nasal swabs were collected every 14 days and humoral responses to full length spike (S) and receptor binding domain (RBD) were assessed. All AMGs were challenged with SARS-CoV-2 B.1.351 (beta variant) on day 56. Viral loads measured every two days by TCID_50_ in nasal washes and bronchial lavage fluid post challenge.

**Results:**

Mucosal immunization with Wuhan-S induced significant increases in serum IgG and IgA responses against the homologous parental lineage, as well as beta, delta, and omicron variants. In nasal samples, Wuhan-S immunization elicited over 500-fold increases in in cross-reactive IgA against multiple variants of concern including delta and omicron. While the beta-S rAd5 vaccine candidate induced enhanced serum IgG responses to homologous S and RBD proteins, this approach resulted in less cross-reactive antibodies to other variants compared to Wuhan-S rAd5 vaccine. Despite the differences in the ability to elicit cross-reactive antibody responses, all vaccinated AMGs challenged with SARS-CoV-2 B.1.351 (beta variant), had a significant reduction in viral titers by TCID_50_ in the nasal passages and reduced viral load in bronchial lavage fluid compared to unvaccinated controls.

**Conclusion:**

These results demonstrate mucosal administration of rAd5 clinical candidate vaccine, Wuhan-S, is immunogenic and offers cross-protective humoral responses in both serum and nasal compartments against a mismatched SARS-CoV-2 challenge virus.

**Disclosures:**

**Becca A. Flitter, PhD, MPH**, Vaxart Inc: Stocks/Bonds **Sarah N. Tedjakusuma, n/a**, Vaxart Inc: Stocks/Bonds **Colin A. Lester, n/a**, Vaxart Inc: Stocks/Bonds **Elena D. Neuhaus, n/a**, Vaxart Inc: Stocks/Bonds **Susan Johnson, PhD**, Vaxart Inc: Stocks/Bonds **Emery G. Dora, n/a**, Vaxart Inc: Stocks/Bonds **Nadine Peinovich, MPH**, Vaxart Inc: Stocks/Bonds **Clara B. Jegede, n/a**, Vaxart Inc: Stocks/Bonds **Sean N. Tucker, PhD**, Vaxart Inc: Stocks/Bonds.

